# Foreign Summary

**Published:** 1839-02-23

**Authors:** 


### FOREIGN SUMMARY.

*A case of Carditis.* By Thos. Salter, Esq.—The author begins by observing upon the extreme rarity of cases of genuine carditis, or inflammation of the muscular substance of the heart, a fact of which the older pathologists, who confounded this affection with pericarditis, do not seem to have been aware. "It does not appear," says the author, "that Corvisart or Laennec ever saw an unmixed case of carditis, nor does Andral give a single instance of the disease." The clearest case of the disease that has ever, in his opinion, been published, is that related by Mr. Stanley, in the seventh volume of the Society's Transactions; and even that presented unequivocal evidence of the co-existence of inflammation of the pericardium.

The patient whose case forms the subject of the present narrative, came under the author's observation eight days before he died, at which time he complained of uneasy sensations in the region of the stomach, and under the sternum, increased by exertion. On applying the ear to the region of the heart, nothing abnormal was discovered. The patient stated that he had first observed the symptoms six weeks before, whilst walking; that the pain was then at the lower part of the chest, inclining to the left side, and that although it did not continue long, it was remarkably severe. A week afterwards he had a similar attack following exertion. The attacks then became more frequent, and even lifting the arm sometimes gave rise to them.

When the author saw him, he was sitting up in bed, being unable to lie down, owing to the great distress in his breathing. He indicated the middle of the sternum as the seat of the pain, which was not lancinating, but of a dull, heavy description. The treatment adopted, including venesection, counter-irritation, and the administration of opium and calomel, failed to procure any alleviation of the symptoms, under which he sank; retaining, however, his mind in a collected state during the entire period of his illness.*

On dissection, the vessels on the bag of the pericardium were distended with red blood. Its reflected layer, especially that covering the left ventricle, also evinced the existence of inflammation which had proceeded to the greatest extent in the part of the membrane attached to the diaphragm, which presented ecchymosed spots resembling purpura hæmorrhagica. The substance of the heart was moderately firm; that forming the left ventricle had almost entirely lost its muscular colour, and pus could be scraped from its cut surfaces. In some parts there were small cavities in the muscular substance, containing pus.—*Lon. Med. Gaz. Jan.* 19.

*Researches on the nature and origin of Pus, its action on the blood, and the distinctive characters between Pus and Mucus.* By M. Mandl.

[Translated for the Medical Examiner, from L'Experience, January 3, 1839.]

My object is not to point out the difference between the pus of an abscess and the mucus of

* In Mr. Stanley's case, alluded to above, so entire was the absence of all symptoms referable to the *heart*, that it was only by dissection ascertained that the patient did not die of disease of the brain.---*Secretary, Med. Chir. Soc.*

the sputa,—it is unnecessary to call to our aid either chemistry or the microscope, when the naked eye alone can decide the question,—thus, we shall not describe the properties of these secretions. But the question is, can we infallibly distinguish mucus from pus, that is to say, from that secretion which is consequent to a wound or a change of texture in a secretory surface? We acknowledge the difficulty we have found in arriving at the desired result. The globules of pus differ in nothing from those of mucus, except in the abundance of globules in the former and the presence of detached epithelium in the latter. Thus, for example, the vaginal mucus is composed almost entirely of shreds of epithelium, as has been remarked by M. Donné, and the globules in it are very few, though the slightest irritation of the vagina causes them to appear. We see in the mucus of the mouth, and especially on the tongue, hardly any thing but the particles of epithelium, constantly renewed by the buccal secretion; but the least catarrh, or the slightest irritation of the digestive passages, produces an increased secretion in the above mentioned parts, and the mucus becomes loaded with globules. The same fact holds true with regard to the sputa, whether suppuration in the lungs may or may not exist,—only in the first case the diffluent tubercular mass produces very small molecules, resembling in all respects the albuminous flocculi usually found in mucous sputa. The microscope confirms, then, what chemistry had already discovered; that is to say, that there is no essential difference in the elementary parts of these secretions. We will endeavour to explain in the following section, the causes of this identity, by showing what we conceive to be the process of secretion.

We have already shown that the globules of pus and those of mucus possess the chemical properties of fibrin. We have seen that the false membranes show a perfectly globular structure. If the secretion from a blister be collected, a matter is seen to coagulate spontaneously, and form a clot in the centre of the liquid. Now, this matter, which we call fibrin, on account of its spontaneous coagulation and insolubility by heat, is composed of globules precisely similar to those of pus. If blood, flowing from a vein, be mingled with the white of an egg, which contains no globules except a few rare molecules, and is agitated with a rod to separate the fibrin, this last is obtained in very soft and easily separated shreds. These particles, examined by the microscope, are composed of the most perfect purulent globules. The fibrin separated from pure blood which has been agitated, proves too compact a mass to be successfully examined by the microscope. Let us recollect, now, what we have said concerning the white mamellated globules we have noticed in the blood. They are, in all respects, similar to the purulent globules, and possess the same chemical properties, so that by a single microscopic observation we cannot decide whether the blood is pure or mingled with pus, for little globules are met with in the purest blood. They have the same form in the different classes of animals, as well as the globules of pus; they do not exist in the circulation; they are found only in blood examined in the object-glass, or in the vessels after death. The blood in the dead subject is found full of these globules, principally in those parts where there has been no formation of clots. Now, all these reasons induce us to conclude, that these globules are the elementary parts of the fibrin which is coagulated; and when these elementary parts in this case are separated, they do not form a coherent mass. This mass contains a very great number of united globules, which cover each other, so that their forms cannot be distinguished, as it happens in the clot of blood.

Now, we say that the globules of pus and of mucus are identical with the white fibrinous globules of the blood. But how shall we account for the origin of the purulent and mucous particles? This is the explanation which our experience has confirmed. The blood, during its circulation, contains the fibrin dissolved in serum. According to the laws of endosmosis and exosmosis, a part of the serum must pass through the walls of the vessels,—the serum, once out of the circulation, leaves the fibrin to coagulate, which it does in its elementary parts, in globules. We have, then, no need of a peculiar membrane for the formation of pus; and we understand why pus and mucus contain all the constituents of the blood, except its colouring matter, which, existing in the globules, cannot pass through the walls of the vessels;—we understand, also, how that, by a longer or shorter retention in the secretory organs, the serum of these different secretions may undergo different changes. By these secretions the fibrin is constantly suffering a loss; and we may infer from the number of globules, that the loss is still greater during suppuration. The experiments we are about to show, may serve to dissipate all doubts on this subject.

If the experiments performed by Müller be repeated,—which consisted in filtering the blood of frogs,—a liquor will be seen to pass through the paper, which, collected on a watch glass, will very readily show small clots floating on its surface. Now, these little fibrinous flocculi, examined with care, are composed of globules, resembling, in all respects, the globules of pus and mucus. Very moderate compression of the particles destroys their elementary composition, and reduces the whole to a shapeless mass. Here, then, we have fibrin composed of globules, obtained by filtering, directly from the blood; now, these globules could not have existed before the coagulation of the fibrin; that is to say, before the serum of the blood had passed through the filtering paper. If it is wished to filter pus through the same paper, whether it be left to itself, or diluted with water to facilitate its filtration, the large globules of pus do not pass. But the globules of fibrin of the same size as those of pus, being found in the filtered liquid, must necessarily be a product of coagulation; for, if they had pre-existed, they would not have been more able to pass than those of pus.

These simple experiments appear to us to confirm satisfactorily our ideas as to the origin of pus and mucus.

---

*University College of London.*—Dr. Elliotson, known formerly as a lecturer, and who has lately acquired not very desirable notoriety for his connection with Animal Magnetism, has resigned the chair of theory and practice, and Dr. Copland, an able writer, has been appointed in his stead.

---

*Extract from a Lecture on the Nervous System.* By Marshall Hall, M. D., January, 1839.—Let me now call your attention to the true spinal, or excito-motory system. I shall, in the first instance, confine myself to a detail of *experiments;* and I think I may therefore assert that what I shall state may be considered as physiologically demonstrated.

If, the head of an animal being removed, you irritate the spinal marrow, those muscles which receive nerves from *below* the point so irritated, are *excited* into a state of contraction. If, instead of irritating the spinal marrow, you irritate a muscular nerve in its course, the muscle or muscles to which this nerve is distributed, are, in like manner, excited into contraction.

Haller, who specially treated of this subject, denominated the power in the nervous system, which is thus called into activity, the *vis nervosa;** the power of contraction in the muscular fibre, he termed its irritability, or the *vis insita.* Of the former, he observes, "Irritato nervo, convulsio in musculo oritur, qui ab eo nervo ramos habet. Irritato vero nervo, multis musculis communi, totive artui, omnes ii musculi convelluntur, qui ab eo nervo nervos habent, *sub* sede irritationis ortos. Denique medulla spinali, irritata, omnes artus convelluntur, qui *infra* eam sedem nervos accipiunt; neque contra artus, qui *supra* sedem irritationis ponuntur." Haller concludes, "Conditio illa in nervo, quæ motum in musculis ciet, *desuper* advenit, sive a cerebro et medulla spinali, *deorsum*, versus extremos nervorum fines propagatur." And "Ut adpareat causam motus a trunco nervi in ramos, non a ramis in truncum venire."† It was a mistake, as M. Flourens has shown, to suppose that this power exists in the *cerebrum.*

This same power is more correctly denominated by Professor Müller, the motorische kraft, or the *vis motoria.* This equally celebrated physiologist treats this subject still more at length, and has laid down the following laws in regard to the mode of action of the motor power:—

"1. The motor power acts only in the direction of the primitive nervous fibres going to muscles, or in the direction of the branches of the nerves; and *never backwards.*

"2. The mechanical or galvanic irritation of a part of a nervous trunk does not excite the motor power of the whole nerve, but only of the isolated part.

"3. A spinal nerve which passes into a plexus, and assists, with other spinal nerves, in the formation of a large nervous trunk, does not impart its motor power to the whole of that trunk, but only to the fibres which it affords in its course from that trunk to the branches.

"4. All nervous fibres act in an isolated manner, from the trunk of a nerve to its ultimate branches."*

M. Flourens denominates this power the "*excitabilité*," and he has shown more distinctly than any preceding physiologist, that it exists in the whole of the medulla oblongata and medulla spinalis, inclusive of the tubercula quadrigemina, but exclusive of the cerebrum and cerebellum; and, of course in the muscular nerves.

These statements convey a true view of the condition of our knowledge on this subject when I first began my researches. The *anatomical limits* and the *course of action* of this motor power were understood to be those which I have thus stated from these celebrated physiologists.

That this statement is true, in reference to the subject of my researches, I am enabled to prove, by detailing an unsuccessful experiment made by Professor Müller.

"I wished," says Professor Müller, "to ascertain whether the last of the spinal nerves, if they were divided at some distance from the spinal marrow, and galvanised, (their roots being still attached to this organ,) would excite convulsive movements in the *anterior* parts, through the medium of the spinal marrow. The results were constant but unexpected.

"Neither the anterior nor the posterior roots occasion, when they alone are acted upon by galvanism, retrograde action into the anterior parts of the animal frame, as of the head. It seems, therefore," adds Professor Müller, "that the fibres of the nerves do not communicate in the spinal marrow."†

Allow me, now, to call your attention to a series of experiments of my own. If, in a turtle, its head having been previously removed, we carefully lay bare the intercostal nerves, and pass across them the galvanic influence, we immediately induce movement in *all* the extremities, *the anterior* and the posterior. If we choose a nerve near the anterior extremities, these are most

* Some writers having been misled, by this expression, to think there is a similarity in the views of Prochaska and my own, I must caution you that this vis nervosa of Haller is *not* the vis nervosa of Prochaska. I can scarcely tell you *what* the vis nervosa of Prochaska is, unless, indeed, it be *every thing.* It is something which is *augmented* in *mania* and in *gout.*

† Elementa Physiologiæ, t. iv. p. 325.

* Handbuch der Physiologie, i. 656; and Dr. Baly's Translation, i. p. 680.

† Handbuch der Physiologie, 1833, t., i. p. 632; Translation, p. 645.

moved; if near the posterior, these, in their turn, are most affected. This experiment, as you will perceive shortly, forms the *basis* of the system of incident, excitor, motor, or excito-motory nerves.

That the source or principle of action in this experiment is identical with that already noticed, as acting in the spinal marrow and in the muscular nerves, is proved by an intermediate experiment. If, in a decapitated turtle, instead of laying bare the intercostal nerve, we denude the spinal marrow, and pass the galvanic influence across its substance, we also excite contractions in the *anterior* as well as the posterior extremities.*

We have thus traced the influence of the "*vis nervosa*," of Haller, the "*vis motoria*," the "*excitabilité*," in a retrograde direction in the spinal marrow itself, and in an incident and retrograde direction in the intercostal nerves and the spinal marrow.

In this latter case we have, I think, a new kind of action, and, physiologically speaking, a new kind of nerve, that is, an *incident motor action*, and an *incident motor nerve.*

May I not affirm that this statement is the pure expression of *facts*, and destitute of *all hypothesis?* for I speak not of *fibres* nor of *filaments*, about which there has been so much discussion; but of an obvious action in an obvious nerve. These two things are as "demonstrated" as any in physiology.

This incident motor nerve thus *demonstrated*, is but one of a *system* of incident nerves, of which I must speak to you. But, before I proceed to that subject, allow me to detain you one moment, to say that what I have just stated is *not* to be found in the admirable works of *Prochaska*, or of *M. Flourens*, or of *any* physiologist with whose labours I am acquainted. Yet it is, as I have stated, the *basis* of that *system of nerves*, and, I may add, of *functions*, to which I have alluded; and it is that basis without which that system could not be satisfactorily established.

I have hitherto spoken of the *trunk* of one of the incident, excitor, or motor nerves. But I must now inform you that the extreme terminations or distributions of these nerves possess the excitor or motor, or, as I have ventured to express it, the excito-motory power, in a much higher degree than their trunk. If, having removed the head of a frog, you divide the integuments along the back, and raise them by means of the forceps, you will observe the trunks of many cutaneous nerves; now, if you irritate these trunks, no movements follow; but, if you irritate the cutaneous textures on which they are distributed, movements of a very energetic character are produced. (See my Memoirs, p. 48, § 21.)

I now proceed with my detail of the series of experiments on the decapitated turtle. If you irritate the nostrils, or the palatine fringes, you excite, through the *trifacial nerve* an act of inspiration. If you irritate the larynx by passing a probe along the trachea, you produce the same effect through the medium of the *pneumogastric* nerve. A similar phenomenon is produced by irritating the trunk of the pneumogastric, or the substance of the spinal marrow, near the points of their division respectively.

Have not these experiments proved, without one word of argument, the existence of *other two incident* nerves possessing *the special excito-motory property?* Do you not perceive the beginnings of *the system of incident excitor nerves* to which I have alluded?

Do you not further perceive that these nerves are not only excitors of muscular action, but, in the case last detailed, of *the act of inspiration?* And do you not now plainly see the application of the "*vis motoria*," the "*excitabilité*," and of the system of incident nerves, through the medium of which it acts, to *physiology?*

This *system* of nerves is further displayed in this diagram and table, and the *extensive class of functions*, for such it is, of which it is the *anatomy*, is displayed in this table.

Until the period of my researches, I believe that the pure motor power of the nervous system had never been applied to physiology,—that the idea of an *incident motor* nerve did not exist; and, consequently, that the system of such nerves, and the special physiology of this system, was totally unknown.

Why do I mention these things? Because it has been eagerly attempted to transfer the credit of what I have done to others, and especially to Prochaska, on the one hand, and to M. Flourens on the other. But I will ask you, whether the idea of an *incident motor* nerve exists in either of these authors? and if not, whether the *system* of such nerves, with their *physiology*, *can* exist in them? In fact, Prochaska goes no further than Whytt; he alludes to *reflex* action, as seen in the *very obvious pathological* phenomena of sneezing, coughing, &c., and then all is confusion; for with these phenomena are associated, as of a *similar* character, the motion of the arm to the head, *said* to take place in apoplexy! the motion of the eye-lids when a person approaches your eye with a finger! the motion of the *heart!* of the *intestines!* &c. It is impossible to argue with persons with such confusion of ideas. And for M. Flourens, he does not make the slightest allusion to an *incident* motor action, or an *incident* motor nerve, or to a *reflex* action; and yet all the functions to which I now refer *are* reflex, and effected by means of such a power, and through the medium of such nerves! On the contrary, M. Flourens states a thousand times in his beautiful work, which is a model of physiological investigation, that respiration, for example, has its *primum mobile* in the medulla oblongata. It *has* its primum mobile in *incident excito-motory nerves.*

Now let me return to this table and diagram.

* A similar experiment was performed both by M. Flourens and Professor Muller; (see Systeme Nerveux, 113; Handbuch, t. i., p. 632, omitted in the Trans.;) but in these experiments the animal was not decapitated; the action of the special motor power could not, therefore be distinguished from the influence of *sensation and volition;* and the experiment is, therefore, *not the same.*

*Anatomy of the true Spinal, or Excito-motory System.*

I. *The Incident, Excitor Branches.*

1. The trifacial, arising from

   1. The eyelashes.

   2. The alæ nasi.

   3. The nostril.

   4. The fauces.

   5. The face.

2. The pneumogastric, from

   1. The pharynx.

   2. The larynx.

   3. The bronchia.

   4. The cardia,—kidney and liver.

3. The posterior spinal, arising from

   1. The general surface.

   2. The glans penis and clitoridis.

   3. The anus.

   4. The cervix vesicæ.

   5. The cervix uteri.

II. *The True Medulla Oblongata and Medulla Spinalis, the Centre of the System.*

III. *The Reflex, Motor Branches.*

1. The trochlearis } Oculi.

2. The abducens }

3. The minor portion of the fifth.

4. The facial, distributed to

   1. The orbicularis.

   2. The levator alæ nasi.

5. The pneumogastric, or its accessory.

   1. The pharyngeal.

   2. The œsophageal.

   3. The laryngeal.

   4. The bronchial, &c.

6. The myo-glossal.

7. The spinal, distributed to the

   1. Diaphragm, and to

   2. The intercostal and } muscles.

   3. The abdominal }

8. The sacral, distributed to

   1. The sphincters.

   2. The expulsors, ejaculators, the fallopian tubes, the uterus, &c.

*All* the nerves represented on this, the left of the table, are *incident motor nerves.* Some of them are *sentient;* but, whether sentient or not, they are demonstrably *motor*, and, whilst they are *motor*, they are *incident.* I beseech you not to allow these two words—these two ideas—to be disjoined in your mind; and there will then be an end of all dispute.

And now let me recall your attention to this table.

*Physiology of the Reflex, Excito-motory System.*

The action—1. Of the eyelids.
- 2. Of the orifices.
  - 1. The larynx;
  - 2. The pharynx.
- 3. Of the ingestion.
  - 1. Of food.
    - 1. In suction;
    - 2. In deglutition.
  - 2. Of air;
  - 3. Of semen.
- 4. Of exclusion.
- 5. Of the expulsors, or of egestion.
  - 1. Of the fæces;
  - 2. Of the urine;
  - 3. Of the semen;
  - 4. Of the fœtus.
- 6. Of the sphincters.

It presents you with the *physiology* which corresponds to the *anatomy.* It presents you with an arrangement of the functions of *ingestion* and *egestion*, of the *orifices* of the *sphincters.* No one has pretended, except in the vaguest manner, and under the shelter of the phrase "*sympathetic actions*," that this extensive view of the subject had been taken before. Professor Müller distinctly states (Trans. p. 803) that the *reflex actions* have a limited place in *physiology.* Prochaska does not name of these functions!! M. Flourens does not name one of them!!! And certainly no one has traced these functions to the *incident* action of the *vis motoria*, in *incident* and purely *moter* nerves, for the very existence of such nerves was unknown. In a word, gentlemen, it was impossible to explain the anatomy or the physiology of *deglutition*, of *inspiration*, of the various *expulsions*, until I published my discoveries of the true spinal marrow, and the excito-motory system of nerves.

What shall I say of the *pathology* of this system? It was impossible that such facts as teething and tetanus, facts as *obvious* as those already mentioned, should have been otherwise than associated with the nerves. How could *the whole closs of spasmodic diseases,—centric, centripetal*, and *centrifugal*, (to use words not my own,) be traced to, and associated with, a part of the nervous system still unknown? One of the most beautiful facts which I shall have to explain to you, is that of the very parts or organs enumerated in the physiology, being precisely those involved in the pathology; so that you have, in many instances, but to recall the former, in order that you may recollect the latter, and in this manner you will be greatly assisted in remembering the *symptoms* of the class of spasmodic diseases.

In conclusion, I must observe that the true spinal or excito-motory system, being the system of ingestion and expulsion, of the orifices and sphincters, is the system of actions on which depend

1. *The preservation of the individual,* and*
2. *The continuation of the species.*

It is in this large and extensive sense that the excito-motory system must be viewed, if we would see its real magnitude and importance. It is in this sense that it will be viewed by the anatomists and physiologists of a future age.

*I must again guard my reader against supposing that I use the first of these phrases in the sense in which Prochaska has used a similar phrase. Prochaska adduces, as an example of what he means, the acts of sneezing, coughing, vomiting, which certainly tend to *remove* what would be injurious, and may be supposed to effect "nostri conservatio."

